# Protocol for analyses of adverse event data from randomized controlled trials of statin therapy

**DOI:** 10.1016/j.ahj.2016.01.016

**Published:** 2016-06

**Authors:** Cholesterol Treatment Trialists' (CTT) Collaboration, C. Reith, L. Blackwell, J. Emberson, B. Mihaylova, J. Armitage, J. Fulcher, A. Keech, J. Simes, C. Baigent, R. Collins

## Abstract

The Cholesterol Treatment Trialists' (CTT) Collaboration was originally established to conduct individual participant data meta-analyses of major vascular events, cause-specific mortality, and site-specific cancers in large, long-term, randomized trials of statin therapy (and other cholesterol-modifying treatments). The results of the trials of statin therapy and their associated meta-analyses have shown that statins significantly reduce the risk of major vascular events without any increase in the risk of nonvascular causes of death or of site-specific cancer, but do produce small increases in the incidence of myopathy, diabetes, and, probably, hemorrhagic stroke. The CTT Collaboration has not previously sought data on other outcomes, and so a comprehensive meta-analysis of all adverse events recorded in each of the eligible trials has not been conducted. This protocol prospectively describes plans to extend the CTT meta-analysis data set so as to provide a more complete understanding of the nature and magnitude of any other effects of statin therapy.

The Cholesterol Treatment Trialists' (CTT) Collaboration meta-analyses of individual participant data from large randomized controlled trials of statin therapy^1^ have shown that statin therapy reduces the risk of major vascular events (ie, myocardial infarction, coronary death, stroke, or coronary revascularization) by approximately one-fifth per millimole per liter (39 milligrams per deciliter) reduction in low-density lipoprotein (LDL) cholesterol, without any increase in the risk of nonvascular causes of death or of site-specific cancer.^2^, ^3^, ^4^, ^5^, ^6^, ^7^ Benefits have been demonstrated in a wide range of people with preexisting vascular disease,^2^, ^4^, ^7^ diabetes,^3^ or other conditions increasing the risk of atherosclerosis, as well as in those with no prior history of vascular disease.^5^ Large randomized trials and meta-analyses of those trials have also established that statin therapy causes a small absolute excess risk of myopathy (usually defined as muscle pain, tenderness, or weakness with creatine kinase [CK] >10 times the upper limit of normal), typically about 1 extra case per 10,000 patient-years of treatment, and an even smaller risk of rhabdomyolysis (typically defined as myopathy associated with renal impairment and/or myoglobinuria, usually accompanied by a CK many multiples [eg, >40 times] greater than the upper limit of normal).^8^ In addition, such studies have shown that statins cause small increases in the incidence of diabetes^10^, ^9^ and, probably, of hemorrhagic stroke (although the overall risk of stroke is reduced).^4^, ^11^ Even in low-risk populations, however, the cardiovascular benefits of statins exceed these hazards.^5^

Treatment guidelines have extended statin therapy recommendations to wider primary prevention populations, such as people who have a 10% or greater 10-year risk of developing cardiovascular disease (UK National Institute for Health and Care Excellence guideline^12^) or those aged 40 to 75 years with a 7.5% or higher risk for myocardial infarction, coronary heart disease death or stroke within 10 years (American College of Cardiology and American Heart Association guideline^13^). This has resulted in public debate about potential adverse effects of statins.^14^ Concern has arisen, chiefly based on reports from nonrandomized observational studies of routine healthcare data, about associations between statin use and higher rates of a wide range of adverse events, including muscle pain (ie, myalgia, as distinct from myopathy) or weakness,^15^, ^16^ hepatic dysfunction,^17^, ^18^ cataracts,^17^, ^19^ depression,^20^ impaired cognition,^21^ sleep disturbance,^22^ and acute kidney injury.^17^, ^23^ There have also been reports from such studies of associations between statin use and lower rates of various nonvascular events, such as cancer,^24^ respiratory conditions,^25^, ^26^, ^27^ fractures,^28^ and Parkinson's disease.^29^, ^30^ However, such associations in nonrandomized observational studies could be due to differences between the people who do and do not take statins in their underlying risks of having particular health outcomes or in the reporting and detection of health outcomes.^31^, ^32^, ^33^, ^34^

Compared with observational studies, the most important methodological strength of randomized controlled trials is that the process of randomization results in groups of patients who differ from each other only by the play of chance with respect to their risks of experiencing all types of health outcome.^31^, ^32^, ^33^, ^35^ In addition, in contrast to observational studies, the ascertainment and definition of health outcomes in the controlled circumstances of a randomized trial are usually systematic and consistent. Furthermore, blinding study treatments through the use of a matching placebo helps to ensure nondifferential assessment of outcomes in the different randomized treatment groups within a randomized trial. Consequently, subject to tests of statistical significance, differences in the rates of health outcomes between the study treatment groups can be attributed causally to the randomly assigned treatment (by contrast with the situation in observational studies).

The previous randomized trials of statin therapy typically collected extensive information on adverse events, which will have been carefully reviewed not only by the trial investigators but also by regulatory authorities (although not necessarily published in its entirety). Many of the larger trials (eg, those recruiting several thousand individuals or more) have had sufficient statistical power on their own to detect any large adverse effects of a statin on particular adverse events (eg, an absolute excess of approximately 10-20 per 1,000 person-years), so it is unlikely that any effects of such magnitude are yet to be identified. However, it is possible that smaller increases or reductions in the risks of adverse events could have been missed by individual trials that would be detectable in a meta-analysis of these trials. For example, despite apparently contradictory^36^, ^37^ but mostly nonsignificant findings in individual randomized trials, recent meta-analyses of the available data have shown that statin therapy is associated with approximately a 10% to 20% proportional increase in the risk for developing diabetes,^10^, ^9^ equating to approximately 1 to 3 additional cases per 1,000 person-years of statin treatment in those trials.

The CTT Collaboration involves the principal investigators and sponsors responsible for conducting around 30 large randomized trials of statin therapy in approximately 200,000 people. For its previous analyses, the outcome data obtained on individual participants in these trials were limited to major vascular events, site-specific cancers, and cause-specific mortality.^1^ As no comprehensive summary of the effects of statins on all recorded adverse events is currently available to prescribers and patients, the present protocol describes plans to seek individual participant data on all of the other adverse events recorded in eligible trials for detailed analyses.

## Methods

### Study eligibility

Randomized trials of statin therapy will be included in the present analyses if they fulfill the original criteria for the CTT Collaboration analyses,^1^ in particular: (i) no confounding with respect to the statin comparisons (ie, no other intended differences in risk factor modification between the randomized treatment groups) and (ii) recruitment of at least 1,000 participants with scheduled study treatment duration of at least 2 years. Table I lists published trials that were eligible and for which there was agreement in principle for data provision at the time this protocol was finalised.

### Data collection

For each eligible trial, the CTT secretariat will seek:

(i) The methods used for ascertaining all adverse events:
  (a) high-level documentation describing the way in which adverse events were sought and recorded (eg, trial protocols);
  (b) blank copies of the case report forms (and any supplementary forms) on which adverse events were to be recorded and detailed descriptions of how information was elicited (eg, by direct questioning for particular symptoms, or unprompted reports) and what was recorded (eg, whether it included symptom severity);
  (c) details of any coding systems used for adverse events (eg, Medical Dictionary for Regulatory Activities [MedDRA], including version number) and of any trial-specific event definitions (eg, for myopathy or diabetes);
  (d) details of any special assessments of particular health outcomes (eg, cognitive function questionnaires, ophthalmic examinations); and
  (e) statistical methods used in assessing adverse events (eg, statistical analysis plan).
(ii) Any tabulations in published articles or available elsewhere (eg, clinical study reports provided to regulatory authorities or previously unpublished analyses held by trialists) of:
  (a) all types of adverse events that were recorded;
  (b) study drug discontinuations and the attributed reasons; and
  (c) any other trial-specific safety outcomes (eg, incidence of raised blood levels of CK or liver transaminases).

The principal investigators and/or sponsors of each trial will also be asked to provide individual participant data (or, where applicable, provide access to such data, and associated data dictionaries via data sharing platforms) on the following:

(i) participant identifiers: individual participants in each trial are to be coded with a unique anonymized identifier (ideally one which allows linkage with any data provided for the previous CTT meta-analyses);
(ii) baseline variables: additional baseline variables (eg, glycated hemoglobin [HbA1c], glucose) will be sought to help in assessing the effects on particular adverse events (eg, onset of diabetes);
(iii) adverse events: each recorded occurrence (ie, not just the first) of all adverse events, and the time since randomization for each adverse event;
(iv) study treatment adherence: the occurrence and timing of, and attributed reasons for, discontinuing study statin/placebo or starting a nonstudy statin;
(v) comedication: information will be sought on the use of drugs at baseline and during trial follow-up that are relevant for particular adverse events (eg, hypoglycemic drugs that may indicate diabetes mellitus; medications that may interfere with statin metabolism);
(vi) laboratory variables: results will be sought for assays that are relevant for particular adverse events (eg, CK for muscle symptoms; liver transaminases for hepatic function; blood glucose or HbA1c for diabetes development or worsening creatinine for renal function); and
(vii) physical parameters: results will be sought for physical measurements that are relevant for particular adverse events (eg, weight in relation to diabetes).

## Analysis plan

The main objective is to assess the proportional and absolute effects of statin therapy on particular adverse events of interest, so that the balance of benefits and harms in specific types of individuals can be determined.

### Outcomes to be assessed

Several types of outcome will be considered, based on current knowledge of the effects of statin therapy in the following hierarchical order:

(i) adverse events that are definitely or probably increased by statin therapy (ie, myopathy, diabetes, and hemorrhagic stroke): analyses of these events will examine the magnitude, timing, and duration of the excess risks both overall and in particular subgroups (eg, diabetes according to baseline HbA1c, hemorrhagic stroke with or without prior stroke) and will also explore how the magnitude of the risks varies according to how an adverse event is defined (eg, biochemical vs clinical diagnosis of diabetes mellitus);
(ii) muscle-related symptoms: analyses of these events will consider muscle pain (ie, myalgia) and weakness separately from myopathy (as defined above^8^) and will explore whether statins increase reported rates of muscle pain of different levels of severity (including, for example, muscle symptoms given as a reason for stopping study treatment);
(iii) other possible effects of statin therapy: this will include adverse events which have been added to some statin drug labels^38^, ^39^ on the basis that there may be a class effect for such events (including cognitive impairment, depression, sleep disturbance, sexual dysfunction, and interstitial lung disease), as well as adverse events for which it has been suggested there may be a reduction with statin therapy (eg, pancreatitis);^40^ and
(iv) all other adverse events recorded in these trials.

### Main and subsidiary analyses

Preliminary information obtained about trial methodological details and tabular data will be used to construct more detailed plans for the combination of adverse event data from each of the trials. Because there will be some heterogeneity between trials with respect to recorded event categories (eg, adverse events, serious adverse events, drug-related adverse events, etc) as well as the type of event coding system (eg, MedDRA, International Classification of Diseases [ICD], etc), it is intended to create analogous event categories and definitions prior to combining trial event data by grouping adverse events into categories based on body systems (eg, as defined by MedDRA “system organ classes” or “high level group terms”), with more detailed examination in other subcategories (eg, using MedDRA “preferred terms” or “standardized medical queries”) in order to create a single analyzable database. This recoding process will be performed prior to unblinding treatment allocation.

The primary analyses will be conducted among blinded trials that examined a statin versus placebo. Subsidiary analyses will be conducted (i) among blinded trials of more versus less intensive statin regimens; and (ii) among trials that assessed statin therapy without a blinded control group (allowing assessment of any reporting biases in unblinded studies).

### Statistical methods

The primary analyses will include all participants who were randomly assigned to the different study treatment groups, irrespective of whether they remained compliant with their allocated study treatment. Such “intention-to-treat” comparisons are used to provide unbiased assessment of moderate effects of a treatment on relatively common outcomes.^35^ However, due to noncompliance with the allocated study treatment, they may underestimate the magnitude of the effect of actually taking it, so estimates of compliance will be used to evaluate the likely effects of full compliance. In addition, for rare outcomes on which statin therapy may be exerting a large relative effect (as is the case with myopathy), “on-treatment” analyses (ie, excluding those known not to be taking their allocated study treatment) will be performed to help increase sensitivity.

In view of the large number of adverse events that will be examined, both uncorrected *P* values and false discovery rate–corrected *P* values will be presented.^41^ In addition, the proportional effects of statin regimens on adverse events in various different subgroups (eg, among different types of participant or with different statin regimens and/or intensity of statin therapy) will be estimated, and compared using standard χ^2^ tests for heterogeneity or, where appropriate, trend. To provide some allowance for multiple subdivisions, overall rate ratios will be reported with 95% confidence intervals (CIs), but all other rate ratios will be reported with 99% CIs. In estimating absolute differences in the rates of adverse events, the overall proportional effect will be applied to underlying absolute rates of the event in different circumstances (unless there is good evidence that the proportional effect differs materially in different circumstances). Assessment of the relevance of any difference in the rate of an adverse event between the treatment groups will consider the consistency of the findings for related events (eg, similar pathology but greater or lesser severity), variations in the proportional and absolute risk of adverse events in particular types of participants, and the timing of any excess risk (ie, immediate or delayed).

Meta-analyses of tabular data on adverse events in the different treatment groups that are obtained in the initial phase of the project will use inverse variance weighted methods for combining 2 × 2 contingency tables based on the observed minus expected (*o*−*e*) statistic and its variance (for binary data) or differences in means for continuous measures.^42^ For meta-analyses of the individual participant data, the methods used for the main analyses will be similar to those used for previous CTT reports, based on the combination of the log-rank (*o*−*e*) and its variance (*v*) in each trial for the first occurrences of a particular adverse event.^1^ The log of the rate ratio (log RR) is calculated as S/V with variance 1/V (and hence with 95% CI of S/V ± 1·96/√V), where S is the sum over all trials of (*o*−*e*) and *V* is the sum over all trials of *v*. In previous reports, the meta-analyses were performed with these (*o*−*e*) and *v* values weighted by the absolute LDL cholesterol difference in each trial at 1 year but, since the main purpose of the present analyses is to assess the effects of statin therapy (rather than of lowering LDL cholesterol), the emphasis of the present meta-analyses will be on unweighted results. When the primary analyses indicate differences between the treatment groups in the rates of first occurrences of some particular adverse event, exploratory analyses will assess whether there is an effect on the subsequent events. SAS (SAS Institute, Cary, NC) and R (www.R-project.org) will be used for analyses.

Analyses of adverse events recorded in approximately 100,000 participants randomized to statin therapy versus placebo would provide excellent statistical power for detecting small absolute differences in the rates of events that are recorded in more than a few percent of participants. For example, with a 5-year adverse event rate among control-allocated participants of 2% (ie, 0.4% per year), the meta-analysis would have >95% power at 2-sided *P* = 0.01 to detect a relative risk of ≥1.2 (Table II), which translates into an absolute 5-year excess of as little as 0.4% (Table III). Moreover, if the 5-year control rate is 10%, then the meta-analysis would have >99% power at 2-sided *P* = 0.01 to detect a relative risk of 1.1 or more (Table II) or an absolute 5-year excess of 1.0% (Table III).

The CTT Collaboration work has received grants from government (UK Medical Research Council and Australian National Health and Medical Research Council) and from charities (British Heart Foundation, Cancer Research UK, and Australian National Heart Foundation) but not from the pharmaceutical industry. Although most of the trials that will contribute to these analyses were supported (at least in part) by research grants from the pharmaceutical industry, most of them were conducted, analyzed, interpreted, and reported independently of all funding sources by academic investigators.

## Data handling and publication policy

The CTT Collaboration is coordinated jointly by the CTT Secretariat in the Clinical Trial Service Unit & Epidemiological Studies Unit in Oxford and the National Health and Medical Research Council Clinical Trials Centre in Sydney. The CTT Secretariat is responsible for collecting and analyzing the data from participating trials on behalf of the CTT Collaboration.

The CTT Secretariat was responsible for the design, drafting and editing of this protocol, with members of the CTT Collaboration being given an opportunity to review and comment on its wording before submission. All members of the CTT Collaboration will have an opportunity to contribute to analyses of the data, interpretation of the results, and drafting of future reports resulting from this project as members of a Writing Committee. As before, these reports will be published in the name of the CTT Collaborative group. Further details of the CTT Secretariat and Collaborators can be found at www.cttcollaboration.org.

## Figures and Tables

**Table I:** Published trials eligible for the CTT collaborative meta-analyses for which there was agreement in principle for data provision at the time this protocol was finalised

Treatment comparison and trial acronym⁎	Treatment comparison (mg/d)†	No. of patients	Median duration of follow-up‡
Statin vs placebo			
Atorvastatin			
ASCOT-LLA	A10 vs placebo	10,305	3.3
CARDS	A10 vs placebo	2838	4.1
ASPEN	A10 vs placebo	2410	4.0
4D	A20 vs placebo	1255	4.0
SPARCL	A80 vs placebo	4731	4.9
Fluvastatin			
ALERT	F40 then 80 vs placebo	2102	5.5
LIPS	F80 vs placebo	1677	3.9
Lovastatin			
AFCAPS/TexCAPS	L20-40 vs placebo	6605	5.2
Pravastatin			
WOSCOPS	P40 vs placebo	6595	4.8
CARE	P40 vs placebo	4159	5.0
LIPID	P40 vs placebo	9014	6.0
PROSPER	P40 vs placebo	5804	3.3
Rosuvastatin			
GISSI-HF	R10 vs placebo	4574	4.2
AURORA	R10 vs placebo	2773	4.6
CORONA	R10 vs placebo	5011	3.0
JUPITER	R20 vs placebo	17,802	2.0
Simvastatin			
SSSS	S20-40 vs placebo	4444	5.4
HPS	S40 vs placebo	20,536	5.4
Subtotal (18 trials)		112,635	4.8§
Statin vs open control or usual care			
ALLIANCE	A10-80 vs usual care	2442	4.7
Post-CABG	L40-80 vs L2 · 5-5	1351	4.3
MEGA║	P10-20 vs usual care	8214	5.0
GISSI-P	P20 vs no treatment	4271	2.0
ALLHAT–LLT	P40 vs usual care	10,355	4.9
Subtotal (5 trials)		26,633	4.4§
More vs less intensive statin therapy			
IDEAL	A40-80 vs S20-40	8888	4.8
PROVE-IT	A80 vs P40	4162	2.1
TNT	A80 vs A10	10,001	5.0
A to Z	S40 then S80 vs placebo then S20	4497	2.0
SEARCH	S80 vs S20	12,064	7.0
Subtotal (5 trials)		39,612	5.1§

⁎ Trial acronyms (in alphabetical order): AFCAPS/TexCAPS, Air Force/Texas Coronary Atherosclerosis Prevention Study; ALERT, Assessment of LEscol in Renal Transplantation; ALLHAT-LLT, Antihypertensive and Lipid-Lowering Treatment to Prevent Heart Attack Trial; ALLIANCE, Aggressive Lipid-Lowering Initiation Abates New Cardiac Events; ASCOT-LLA, Anglo-Scandinavian Cardiac Outcomes Trial-Lipid Lowering Arm; ASPEN, Atorvastatin Study for Prevention of Coronary Heart Disease Endpoints in Non-Insulin-Dependent Diabetes Mellitus; A to Z, Aggrastat to Zocor; AURORA, A Study to Evaluate the Use of Rosuvastatin in Subjects on Regular Hemodialysis: An Assessment of Survival and Cardiovascular Events; CARDS, Collaborative Atorvastatin Diabetes Study; CARE, Cholesterol And Recurrent Events; CORONA, Controlled Rosuvastatin Multinational Trial in Heart Failure; 4D, Die Deutsche Diabetes Dialyse Studie; GISSI-HF, Gruppo Italiano per lo Studio della Sopravvivenza nell’Insufficienza cardiaca; GISSI-P, Gruppo Italiano per lo Studio della Sopravvivenza nell’Infarto Miocardico Prevenzione; HPS, Heart Protection Study; IDEAL, Incremental Decrease in End Points Through Aggressive Lipid Lowering; JUPITER, Justification for the Use of Statins in Prevention: an Intervention Trial Evaluating Rosuvastatin; LIPS, Lescol Intervention Prevention Study; LIPID, Long-term Intervention with Pravastatin in Ischaemic Disease; MEGA, Management of Elevated Cholesterol in the Primary Prevention Group of Adult Japanese; Post-CABG, Post-Coronary Artery Bypass Graft; PROSPER, PROspective Study of Pravastatin in the Elderly at Risk; PROVE-IT, Pravastatin or Atorvastatin Evaluation and Infection Therapy; SEARCH, Study of the Effectiveness of Additional Reductions in Cholesterol and Homocysteine; SPARCL, Stroke Prevention by Aggressive Reduction in Cholesterol Levels; SSSS, Scandinavian Simvastatin Survival Study; TNT, Treating to New Targets; WOSCOPS, West of Scotland Coronary Prevention Study.

† Statins tested: A, atorvastatin; F, fluvastatin; L, lovastatin; P, pravastatin; R, rosuvastatin; S, simvastatin.

‡ Estimated using Kaplan-Meier method with patients censored at their date of death.

§ Weighted by trial-specific variances of observed log-rank (*o* − *e*) for major vascular events.

║ Includes 382 randomized patients who were excluded from the original publication.

**Table II:** Approximate statistical power (2-sided α = 0.01) among 100,000 participants randomized between statin and placebo to detect relative risks of 1.05, 1.1, 1.2, or 1.3 with absolute 5-year control rates of 2%, 5%, 10%, or 20%

5-y event rate in control group	Hypothetical relative risk associated with allocation to statin therapy			
1.05	1.1	1.2	1.3
Power at 2-sided α = 0.01			
2%	7%	36%	96%	>99%
5%	22%	83%	>99%	>99%
10%	51%	>99%	>99%	>99%
20%	91%	>99%	>99%	>99%

The above power estimates are based on a test of the observed odds ratio under each scenario, which will be slightly larger than the relative risks but will correspond exactly to the relative risks given the control event rate in each case. Odds ratios and relative risks are similar when outcomes are rare but become more different as outcome rates increase (eg, when the 5-year control event rate is 20%, the relative risks of 1.05, 1.1, 1.2, and 1.3 correspond to odd ratios of 1.06, 1.13, 1.26, and 1.41, respectively).

**Table III:** Five-year absolute excess risk under hypothetical relative risks shown in Table II

5-y event rate in control group	Hypothetical relative risk associated with allocation to statin therapy			
1.05	1.1	1.2	1.3
Absolute difference in event rate associated with risk ratio			
2%	0.1%	0.2%	0.4%	0.6%
5%	0.25%	0.5%	1.0%	1.5%
10%	0.5%	1.0%	2.0%	3.0%
20%	1.0%	2.0%	4.0%	6.0%

## Writing committee

Reith C., Blackwell L., Emberson J., Mihaylova B., Armitage J., Fulcher J., Keech A., Simes J., Baigent C., Collins R.
